# Pulse respiration quotient as a measure sensitive to changes in dynamic behavior of cardiorespiratory coupling such as body posture and breathing regime

**DOI:** 10.3389/fphys.2022.946613

**Published:** 2022-12-23

**Authors:** Zoran Matić, Aleksandar Kalauzi, Maximilian Moser, Mirjana M. Platiša, Mihailo Lazarević, Tijana Bojić

**Affiliations:** ^1^ Biomedical Engineering and Technologies, University of Belgrade, Belgrade, Serbia; ^2^ Department for Life Sciences, Institute for Multidisciplinary Research, University of Belgrade, Belgrade, Serbia; ^3^ Chair of Physiology, Medical University of Graz, Graz, Austria; Human Research Institute, Weiz, Austria; ^4^ Institute of Biophysics, Faculty of Medicine, University of Belgrade, Belgrade, Serbia; ^5^ Department for Mechanics, Faculty for Mechanical Engineering, University of Belgrade, Belgrade, Serbia; ^6^ Department of Radiation Chemistry and Physics, “VINČA” Institute of Nuclear Sciences - National Institute of Thе Republic of Serbia, University of Belgrade, Belgrade, Serbia

**Keywords:** heart rate, autonomic nervous system, homeodynamics, pulse-respiratory quantization, vagal activity, sympathetic, respiratory sinus arrhythmia

## Abstract

**Objective:** In this research we explored the (homeo)dynamic character of cardiorespiratory coupling (CRC) under the influence of different body posture and breathing regimes. Our tool for it was the pulse respiration quotient (PRQ), representing the number of heartbeat intervals per breathing cycle. We obtained non-integer PRQ values using our advanced Matlab^®^ algorithm and applied it on the signals of 20 healthy subjects in four conditions: supine position with spontaneous breathing (Supin), standing with spontaneous breathing (Stand), supine position with slow (0.1 Hz) breathing (Supin01) and standing with slow (0.1 Hz) breathing (Stand01).

**Main results:** Linear features of CRC (in PRQ signals) were dynamically very sensitive to posture and breathing rhythm perturbations. There are obvious increases in PRQ mean level and variability under the separated and joined influence of orthostasis and slow (0.1 Hz) breathing. This increase was most pronounced in Stand01 as the state of joint influences. Importantly, PRQ dynamic modification showed greater sensitivity to body posture and breathing regime changes than mean value and standard deviation of heart rhythm and breathing rhythm. In addition, as a consequence of prolonged supine position, we noticed the tendency to integer quantization of PRQ (especially after 14 min), in which the most common quantization number was 4:1 (demonstrated in other research reports as well). In orthostasis and slow breathing, quantization can also be observed, but shifted to other values. We postulate that these results manifest resonance effects induced by coupling patterns from sympathetic and parasympathetic adjustments (with the second as dominant factor).

**Significance:** Our research confirms that cardiorespiratory coupling adaptability could be profoundly explored by precisely calculated PRQ parameter since cardiorespiratory regulation in healthy subjects is characterized by a high level of autonomic adaptability (responsiveness) to posture and breathing regime, although comparisons with pathological states has yet to be performed. We found Stand01 to be the most provoking state for the dynamic modification of PRQ (cardiorespiratory inducement). As such, Stand01 has the potential of using for PRQ tuning by conditioning the cardiorespiratory autonomic neural networks, e.g., in the cases where PRQ is disturbed by environmental (i.e., microgravity) or pathologic conditions.

## Introduction

During physiological regulation, the heart and the lung produce specific rhythms that, recorded by biomedical instrumentation, can be viewed as signals of biological oscillations (fluctuations). Modern research has observed that biological oscillations are characterized by high temporal variability and mutual interaction (coupling). Also, it turned out that these characteristics, especially as found in heart rhythm and breathing are of fundamental importance for health (Moser et al., 2006 a; b, 2008) and that, in a sense, they are very sensitive to changes in physical and physiological conditions. Body posture and breathing regime changes are a kind of perturbations that induce different physical and physiological conditions of cardiorespiratory functioning in the human organism which has adapted to these perturbations through subtle adjustments of the mean values of internal biorhythms (homeostasis) and its variability and complexity characteristics (homeodynamics) (Ernst 2014, 2017; Matić et al., 2020) mostly by mediation of the autonomic nervous system (ANS). Studies of the influence of orthostasis and especially slow breathing on the ANS regulation have become very relevant in recent years, both in fundamental biomedical research and in clinical practice. From scientific evidence, it has been concluded that the heart rhythm in supine position is characterized by a slight predominance of parasympathetic (vagal) activity (Levy and Martin 1996; Malliani 2005; Mclachlan et al., 2010). Conversely, in the standing position, sympathetic predominance is manifested (Levy & Martin, 1996; Srinivasan et al., 2002; Srinivasan et al., 2002; Maggioni et al., 2020). Slow breathing at the rate of 0.1 Hz (6 breaths per min) has been shown to produce resonant effect on different physiological rhythmicity (brain activity (Hinterberger et al., 2019), mental functions (Schwerdtfeger et al., 2020), heart rate variability (Russo, Santarelli and O’Rourke 2017), blood pressure (Russo, Santarelli and O’Rourke 2017; Brenner et al., 2020), etc) and even hematocrit (Schneditz et al., 1990). Mainly, the resonant effect of slow breathing refers to increase in temporal variability which is usually reflected in the rise of standard deviation (variance or total power of spectral density) (Force of The European Society of Cardiology & North American Society of Pacing, 1996). Overall, coupling and resonance that partake in between physiological rhythms increase variability of rhythms themselves (Dick et al., 2014). Evidence for significant increase of heart rate *variability* (HRV) induced by slow breathing near 0.1 Hz frequency is given in many recent studies (Zautra et al., 2010; Lin et al., 2014; Steffen et al., 2017; Blum et al., 2019; Lehrer et al., 2020). This phenomenon of rise in HRV through slow breathing is also called ‘cardiac coherence’ (West, 2010; Shaffer et al., 2014; McCraty, 2017), and the technique itself “HRV biofeedback” (Lehrer et al., 2013). Regarding previous research on this topic, it has been generalized that slow (controlled) breathing can be used for conscious regulation enhancement of emotional, cognitive and physiological performances (Boyadzhieva and Kayhan, 2021).

Besides homeodynamics, breathing and heart rhythm interact mutually in a very dynamic manner, forming an integrated physiological behavior called “cardiorespiratory coupling” (CRC) (Kralemann et al., 2013; Dick et al., 2014). Both CRC and homeodynamics might be enhanced by the vagal activity increase induced by slow breathing (Russo, Santarelli and O’Rourke 2017; Matić et al., 2020).

There are various techniques for exploration of CRC. Among them, pulse respiration quotient (PRQ) was reaffirmed as a very elegant and powerful tool by which useful information on ANS regulation patterns can be obtained (Moser et al., 1995; Edelhäuser et al., 2013; Scholkmann et al., 2019; Scholkmann and Wolf 2019; Grote et al., 2021). In earlier research (at the beginning of the 20th century), PRQ was calculated approximately, as integer values (Coleman, 1920). Except for the investigation of Edelhäuser et al. in which temporal phase coordination between heart beats and breathing was calculated (Edelhäuser et al., 2013), due to the lack of greater precision in the automatic calculation of PRQ, the temporal variability of PRQ remained less researched and interpreted compared to the heart rate (temporal) variability (HRV). Claims that the PRQ can capture reactivity and adaptability of cardiovascular and cardiorespiratory systems to internal and external situations and requirements, that it contains information on “cardio-respiratory adaptability due to change of state” and “the overall current state of human physiology in general” (Scholkmann and Wolf 2019) were based on changes in mean PRQ and should be supplemented by an assessment of PRQ temporal variability. To achieve this, instead of “determining PRQ by averaging HR and BR (breathing rate) measurements” (Scholkmann and Wolf 2019), it is necessary to perform PRQ determination using an algorithm for continuous calculation of heart beat intervals (RRI) per each real-time respiratory cycle. In this paper we focused our analysis on the HR:RR = HR:1 relation, due to the robustness of this type of synchronization (Kotani et al., 2002; Bartsch et al., 2007). In this way, a PRQ signal with high time resolution is obtained. Fluctuations in PRQ signals could be shown to be sensitive to changes in physiological states such as body posture (supine *versus* standing position) and breathing regime (spontaneous *versus* controlled slow breathing). Slow breathing (0.07–0.16 Hz, i.e., 4-10 breaths per minute) influences the PRQ not only by increasing its average value due to the direct effect on the ratio (quotient) of heart and breathing rates, but also by the effect on respiratory sinus arrythmia (Kotani et al., 2002; Russo, Santarelli and O’Rourke 2017).

The basic idea of ​​this research was to examine separate and joint (synergetic) influences of body posture (supine position and standing) and breathing regime (spontaneous and slow-paced 0.1 Hz) on the temporal variability patterns of PRQ. We hypothesized that ANS regulation is responsible for the primary features of PRQ oscillations. Confirmation of this hypothesis was obtained through linear statistic parameters of PRQ. Our aim was to extract information on the state of responsiveness and adaptability of the ANS by dynamic analysis of PRQ in four physiological conditions.

## Methods

Our research included, in detail, reproducible conditions and procedures (maneuvers) in which signal acquisition, application of advanced algorithms for calculating linear characterizing parameters, statistic comparison of results and their systematic physical and physiological interpretation were performed. So, this protocol of simultaneous recording of ECG and breathing signal was applied on 20 healthy subjects in four physiological states (supine position with spontaneous breathing, standing with spontaneous breathing, supine position with 0.1 Hz breathing and standing with 0.1 breathing) and performed under controlled laboratory conditions by means of the Biopac MP100 system (Biopac System, Inc., Santa Barbara, CA, United States; AcqKnowledge 3.91 software). For further details of the study protocol please refer to Matić et al., 2020.

### Data processing

In order to obtain RR intervals (RRI) and breath-to-breath intervals (BBI) it is necessary to detect the R peaks in the ECG recording and the onset of the breathing cycles in the breathing signal. We did that automatically by using the Pick Peak tool of Origin software (Microcal, Northampton, MA, United States). After that we calculated BB and RR intervals as difference between successive time coordinates of R peaks or the onset of the breathing cycle B (minima) according to the expression:

X(i)=Pk(i+1)−Pk(i) , where

Pk(i) - time coordinates of detected RR peaks or B minima Pk (i+1) - time coordinates of subsequently detected RR peaks or B minima

X(i) stands for either of two intervals, i.e., RRI(i) or BBI(i) , with respect to the type of the signal (ECG or breathing).

Pulse-respiration quotient (PRQ; RRI:BBI=m:1) might be obtained in two ways: the first is a simple manual technique of palpation the pulse (or by visual follow up) and counting how many heart beats correspond to each breathing cycle (Coleman, 1920). This is not sufficiently precise for modern scientific evaluations. The second approach is by recording both the ECG and the breathing signals, automatic calculation of RR intervals (RRI) and breath-to-breath intervals (BBI), and calculating the number of RRI per BBI. Next, if RRI and BBI are expressed as counts per minute, we get instantaneous heart rate (HR) and breathing rate (BR) according to the following expressions:

$$HR=\frac{60}{RRI}\;\left[heartbeats/\min\right]$$

$$BR=\frac{60}{BBI}\left[\left(inspiration+expiration\right)\;/\min\right]$$

Then, the instantaneous PRQ is derived as the ratio between HR and BR, or reciprocally BBI and RRI:

$$PRQ=\frac{HR}{BR}=\frac{BBI}{RRI}$$

**FIGURE 1:**
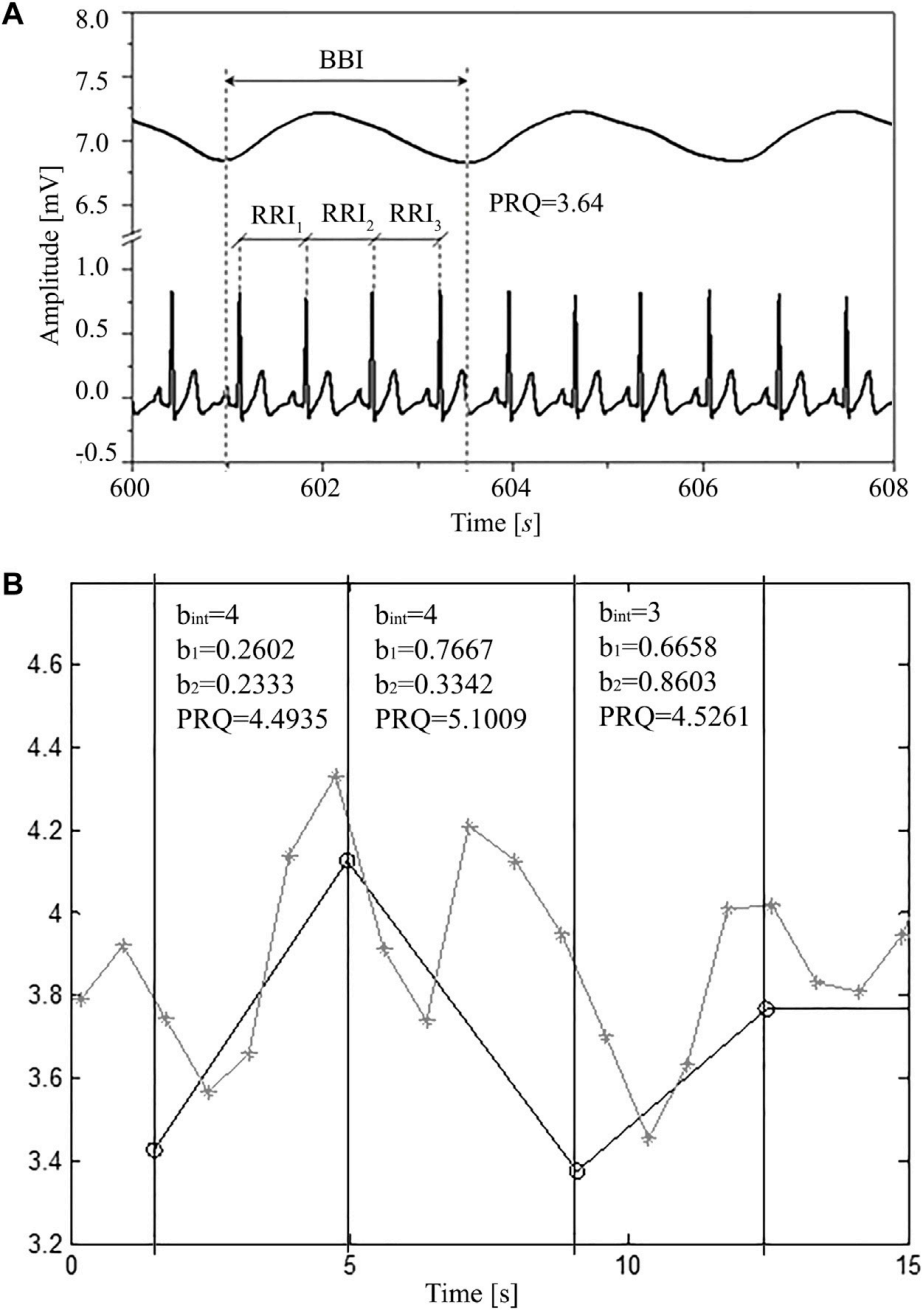
**(A)** A segment of simultaneously recorded signals (8 s selected from a total of 1200 s): breathing and ECG in one subject in a supine position with spontaneous breathing; where: RRI—time interval between two adjacent R peaks of the ECG signal, BBI—breathing interval, calculated as the time difference between onsets of two successive inspirations (respiratory cycle), PRQ—pulse respiration quotient (number of heartbeats per respiratory cycle). **(B)** Obtaining PRQ from signals derived as rr and intervals. Gray line with crosses: rr intervals (ordinates) vs. rr onset time (abscissa). Black line with circles: BB intervals (ordinate) vs. BB onset time (abscissa). Black vertical lines and text boxes with parameter values are added for better explaining the principle behind obtaining PRQ from rr (RRI) and BB (BBI) intervals. Each PRQ value is a sum of the integer number of whole rr intervals (b_int_) and the non-integer parts of rr intervals (b_1_ and b_2_) that fall within one BBinterval (breathing cycle). Thus, PRQ = b_int_ + b_1_+b_2_.

The principle of average PRQ (RRI:BBI = *m*:1) determination is simple: by dividing HR with BR its average value across the whole signal is obtained. However, if we want to explore, as mentioned, its dynamical behavior, precise counting of all RRI that appear within each breathing cycle is needed. Figure 1 illustrates this general principle of PRQ calculation.

In order to automatically calculate (count) the number of RR intervals per respiratory cycle, it is necessary to use an algorithm according to the following procedure (explained for one breathing interval as an example). Suppose that i and e denote the time occurrences (beginnings) of inspiriums and expiriums, respectively, while r marks the occurrences of ECG R-peaks and that they were arranged in the following order:

Respiration e……i1………e………………..i2……

R peaks r0……r1…r2 …….r3….r4.…..r5….r6

Sequential number of 1 2 3 4

Sequential number of RR intervals

First, we count the integer number of whole RR intervals (b_int_) that fall between i1 and i2. In this case there are three: r2−r1, r3−r2, r4−r3 .

Then we add parts of the boundary RR intervals that belong to (i1, i2) breathing interval, as non-integer parts of the PRQ:

b1=(r1−i1)/(r1−r0),and b2=(i2−r4)/(r5−r4)

So, the total instantaneous PRQ value, belonging to (i1, i2) breathing interval, would be

PRQ(i1,i2)=bint+b1+b2=3+b1+b2

In this way we obtain an exact non-integer count, without any averaging.

As well, with this approach no information is wasted, as the total number of RR intervals is conserved throughout the recording and boundary RR intervals are precisely shared between adjacent breathing intervals. For instance, in this particular case, “b2” part of (i1, i2) and “b1” part of (i2, i3) add up to form the whole r4, r5 interval, *etc.*

An example of this procedure applied for obtaining a few quotients from RRI and BBI signals is given in Figure 1.

**FIGURE 2:**
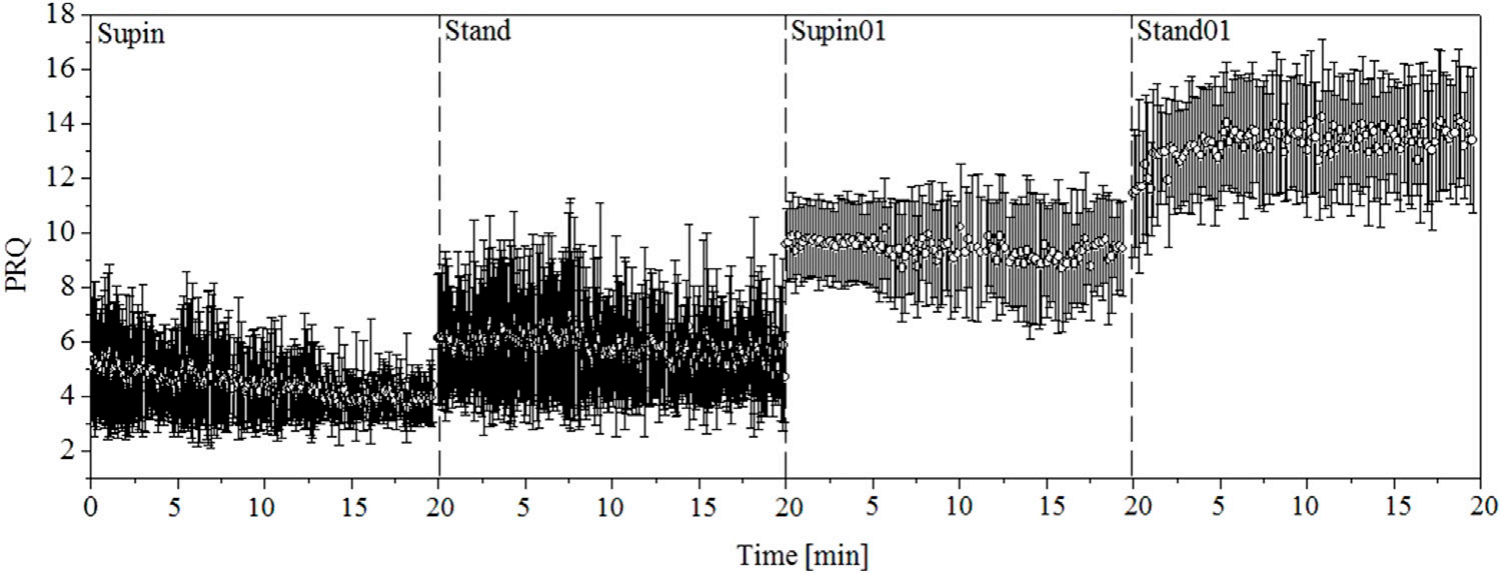
One-minute PRQ mean values and variabilities for 20 subjects. Supin—supine position with spontaneous breathing, Stand—standing with spontaneous breathing, Supin01—supine position with slow (0.1 Hz) breathing, Stand01—standing with slow (0.1 Hz) breathing.

When we apply this algorithm to the whole signals of RRI and BBI, we get a new PRQ signal with continuous precise calculation of its values. After visual presentation of PRQ in the four investigated states (Figure 2), it is clear that PRQ has its own variability, the new information calculated from the variability of RRI and BBI.

In order to obtain additional information about a possible quantization of the values in whole number ratios, we plotted the distributions of PRQ variability data by the means of the histograms and probability density estimation (PDE) profiles. Histograms were calculated using Origin program and PDE profiles by an algorithm created in Matlab 2010a (MathWorks Inc., Natick, MA01760–2098, United States). For a more detailed procedure about the generation of PDE profiles please consult the reference Kalauzi et al. (2012). Comparison of PDE profiles was done by means of Kullback-Leibler divergence (D_KL_) (Kullback and Leibler, 1951). We performed calculation of D_KL_ using Matlab (2010a). In general, D_KL_ (familiar also as relative entropy) is a measure that starts from one distribution, q(x), and calculates how much the new distribution, p(x), is unexpectedly different from the old one, i.e., how much new information it brought in relation to expectations:

$$D_{KL}\left(p\|q\right)=\int_{-\infty }^{\infty }P\left(x\right)\log\left(\frac{P\left(x\right)}{q\left(x\right)}\right)dx$$

We used this relative entropy measure to compare PRQ ensembles in the states of body posture and breathing regime changes and we used supine position as a reference state.

### Statistics

The obtained results were collected and saved in databases created using SPSS 19 (Statistical Package for the Social Sciences 19, IBM, New York, United States). Statistical analysis was then performed using the SPSS 19 tools. Initially, we checked the normality (Gaussian) distribution of the obtained results for each parameter by visual methods (histograms (frequency distribution, tree-leaf plot, stem plot, box plot), PP graph (probability-probability plot) and KK (quantile-quantile graph)) and Shapiro-Wilk normality test. Both visual inspection and Shapiro-Wilk normality test of the PRQ parameters in 20 subjects confirmed that our data did not have a normal (Gaussian) distribution, so we applied the non-parametric Kruskal Wallis test and the post-hoc Man Whitney test with Bonferroni’s correction for multiple measurements to compare all samples (Table 2).

## Results

Using the previously explained method we obtained the PRQ signals in our four investigated states and performed statistical tests for comparison. Figure 2 gives a visual impression of these signals. From the results calculated for 20 subjects, we made a 1-min horizontal mean values estimation of the PRQ parameter in each state. After that, we plotted these mean values with their standard deviations as error bars. In signals plotted in this way, the influence of body posture and breathing regime is notable.

Beside visual presentation, mean and standard deviation values of RRI, BBI and PRQ signal are reported in Table 1. Results for RRI and BBI are given here in order to be compared with PRQ as a new quantity.

**TABLE 1:** RRI, BBI and PRQ (mean ± SD) for 20 healthy subjects in four physiological states; Supin—supine position with spontaneous breathing, Stand—standing with spontaneous breathing, Supin01—supine position with slow (0.1 Hz) breathing, Stand01—standing with slow (0.1 Hz) breathing (adopted from Matić et al., 2020 and completed by PRQ data for comprehensive analysis).

Parameter	Supin	Stand	Supin01	Stand01
mRRI [s]	0.9937 ± 0.1377	0.7263 ± 0.1021	1.0592 ± 0.1257	0.7480 ± 0.0867
sdRRI [s]	0.0621 ± 0.0237	0.0465 ± 0.0175	0.0905 ± 0.0347	0.0702 ± 0.0225
mBBI [s]	4.55 ± 1.45	4.56 ± 1.78	10.0605 ± 0.1942	9.9676 ± 0.1466
sdBBI [s]	0.89 ± 0.61	1.09 ± 1.35	1.4235 ± 0.9437	1.0313 ± 0.4060
mPRQ	4.8118 ± 1.6659	6.3854 ± 2.4308	9.4144 ± 1.2062	13.4761 ± 1.6591
sdPRQ	1.1438 ± 0.6673	1.9374 ± 1.7321	1.3932 ± 0.7117	1.5369 ± 0.5344

In Table 2 we present the results of statistical analysis of the quantities presented in Table 1, for the 20 subjects. Because the data did not display Gaussian distribution (which was confirmed by visual inspection and Shapiro-Wilk normality test), we chose to apply the non-parametric Kruskal Wallis test for comparison of the results. We found a significant state dependent change compared to supine position values (as the baseline) by Mann-Whitney test with a Bonferroni correction of significance for multiple permuted measurements (p*m < 0.05, for m = 3, where m is the number of comparisons)^1^. From Table 2, we can see that orthostasis and slow breathing produce significant changes in RRI, BBI and PRQ.

**TABLE 2:** Change of RRI, BBI and PRQ under different conditions (Post hoc Mann-Whitney test for independent samples with Bonferroni corrected *p*-value (*p*-value ∙ m < 0.5, for m = 3, where m is the number of comparisons) after Kruskal–Wallis test for multiple comparisons for 20 healthy subjects; direction of change: ↓ - decrease; ↑ - increase); Bold font indicates statistically significant changes (p value less than 0.05); gray color of font indicate a value close to 0.5 which could be regarded as a statistically significant change. (Adopted from Matić et al., 2020 and completed by PRQ data for comprehensive analysis).

Parameter	Supin-Stand	Supin-Supin01	Supin-Stand01
mRRI [s]	**0.001↓**	0.306	**0.001↓**
sdRRI [s]	0.072↓	**0.021↑**	0.831
mBBI [s]	>0.99	**-**	**-**
sdBBI [s]	>0.99	**-**	**-**
PRQ	**0.000↑**	**0.000↑**	**0.000↑**
sdPRQ	0.141	0.204	**0.039**↑

Table 3 is given for a more detailed view on temporal behavior of PRQ under the influence of body posture and breathing regime. It was obtained by calculation of minute values of PRQ for each subject. Then, its horizontal mean value with corresponding standard deviations was estimated for the whole set of 20 subjects. Thus, temporal changes of all 20 PRQ minute values could be observed in each of the four states.

**TABLE 3:** Minute values of PRQ parameters (mean ± standard deviation), averaged for 20 subjects in four states. Supin—supine position with spontaneous breathing, Stand—standing with spontaneous breathing, Supin01—supine position with slow (0.1 Hz) breathing, Stand01—standing with slow (0.1 Hz) breathing.

Time (min)	PRQ_Supin_ (--)	PRQ_Stand_ (--)	PRQ_Supin01_ (--)	PRQ_Stand01_ (--)
1	5.26383 ± 2.31472	5.95709 ± 2.20978	9.63582 ± 1.46697	11.79866 ± 2.49239
2	5.013 ± 2.29122	5.98738 ± 2.18034	9.71613 ± 1.4695	12.69322 ± 1.97467
3	4.94698 ± 2.09833	6.15654 ± 2.59441	9.58971 ± 1.40561	12.705 ± 2.0336
4	4.78723 ± 1.64101	6.16053 ± 2.71091	9.68669 ± 1.58039	13.05603 ± 2.15277
5	4.65407 ± 1.58092	6.21829 ± 2.75615	9.62924 ± 1.50201	13.18461 ± 2.10297
6	4.97842 ± 2.18362	6.1176 ± 2.57549	9.58766 ± 1.74327	13.57818 ± 2.10853
7	4.78919 ± 2.17825	6.19114 ± 2.61454	9.17507 ± 1.90426	13.49438 ± 2.32806
8	4.72043 ± 1.93314	6.32685 ± 2.93612	9.42092 ± 1.84727	13.3066 ± 2.15415
9	4.51035 ± 1.60624	5.96568 ± 2.59158	9.37971 ± 1.92135	13.57922 ± 2.25175
10	4.40756 ± 1.51257	5.87847 ± 2.20089	9.35339 ± 1.98915	13.62667 ± 2.2694
11	4.41325 ± 1.60696	5.74063 ± 2.02265	9.56773 ± 2.30129	13.4747 ± 2.36701
12	4.34052 ± 1.2613	5.60282 ± 1.99261	9.46898 ± 1.92909	13.35791 ± 2.21631
13	4.41653 ± 1.54333	5.82723 ± 2.04347	9.18351 ± 2.05129	13.40357 ± 2.06937
14	4.11025 ± 0.97724	5.52863 ± 1.87425	9.1031 ± 2.43961	13.55143 ± 2.27799
15	4.00185 ± 1.04793	5.59959 ± 1.8856	9.08627 ± 2.03071	13.40078 ± 2.24055
16	3.96592 ± 0.92141	5.58987 ± 2.01121	8.97489 ± 2.29802	13.51484 ± 2.053
17	4.07595 ± 0.98849	5.529331.74282	9.32683 ± 1.88533	13.42076 ± 2.21723
18	4.0416 ± 0.88411	5.5067 ± 1.74678	9.47288 ± 1.80233	13.45431 ± 2.49363
19	3.83594 ± 0.95124	5.81484 ± 2.26019	9.33528 ± 1.77989	13.71134 ± 2.24221
20	3.94859 ± 0.90775	5.50322 ± 1.82887	9.24888 ± 2.03297	13.57815 ± 2.227

**FIGURE 3:**
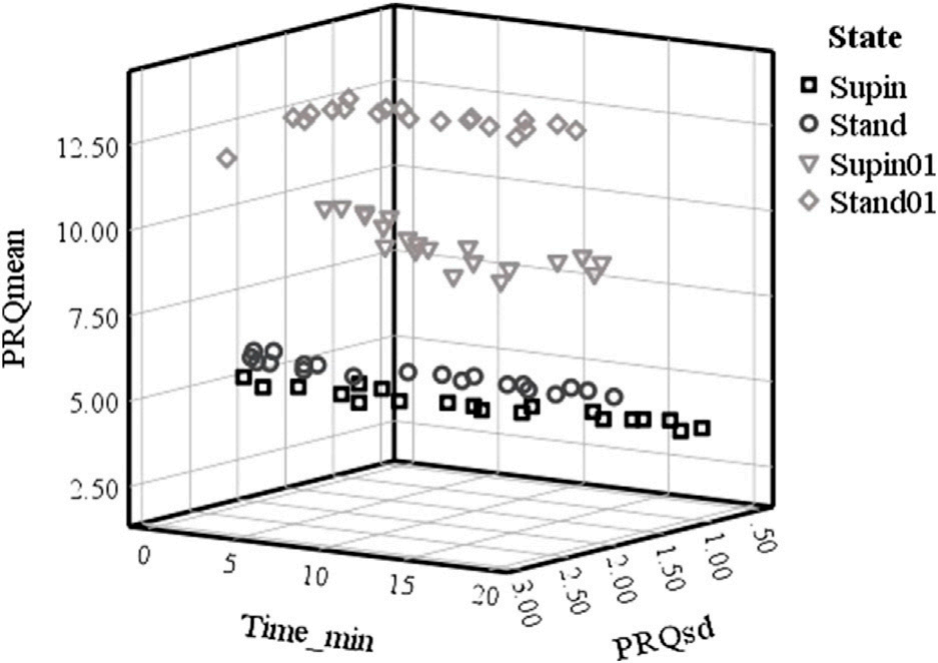
Plot of PRQmean versus PRQsd minute values in time in four physiological states in 20 subjects. Supin—supine position with spontaneous breathing, Stand—standing with spontaneous breathing, Supin01—supine position with slow (0.1 Hz) breathing, Stand01—standing with slow (0.1 Hz) breathing.

Based on Table 3, we plotted minute mean values *versus* standard deviation of PRQ (PRQmean, PRQsd, respectively), which is shown on Figure 3. Figure 3 data allows the discrimination of physiological states. It is obvious that mean vs. std dev points create specific clusters of values from which Supin, Stand, Supin01, Stand01 could be recognized as separate, well defined states.

Clustering of PRQmean *versus* PRQsd minute values *in time* (Figure 3) was as distinctive as the clustering of PRQmean *versus* the average PRQsd minute values. These data support the conclusion that PRQ is regulated constantly and in a stable way during the whole course of all 20-min session but in a state-specific manner. The PRQ state dependent regulation is not, though, robustly regulated, but constantly on at least a 1 min (resolution of our analysis) regular basis. This conclusion, evident from Figure 3, is supported by the minute-by-minute PRQmean *versus* PRQsd Supin and Stand relations. These relations, mutually similar, follows quasi-linear, 3D “cigarette-shaped” regimes of quasi-constant PRQmean coupled to very variable PRQsd. The more dynamical states like Supin01 and Stand01 are mutually similar in shape, 3D “boomerang shaped” PRQmean *versus* PRQsd relation, that represent coupled quasi-constant values of PRQmean with quasi-constant values of PRQsd. (Figure 3). On the basis of the 3D shapes of PRQmean vs. PRQsd relation it is clear that Supine and Stand belong to the one “family”, while Supine01 and Stand01 belong to another “family” of the PRQ regulating regimes. We can reasonably assume that, in our experimental design, the shift from one “family” to another “family” of regulating regimes determines the breathing regime.

Finally, we plotted state-specific probability density estimation (PDE) profiles of PRQ values which demonstrate that different states have specific distributions and accumulation of PRQ values.

**FIGURE 4:**
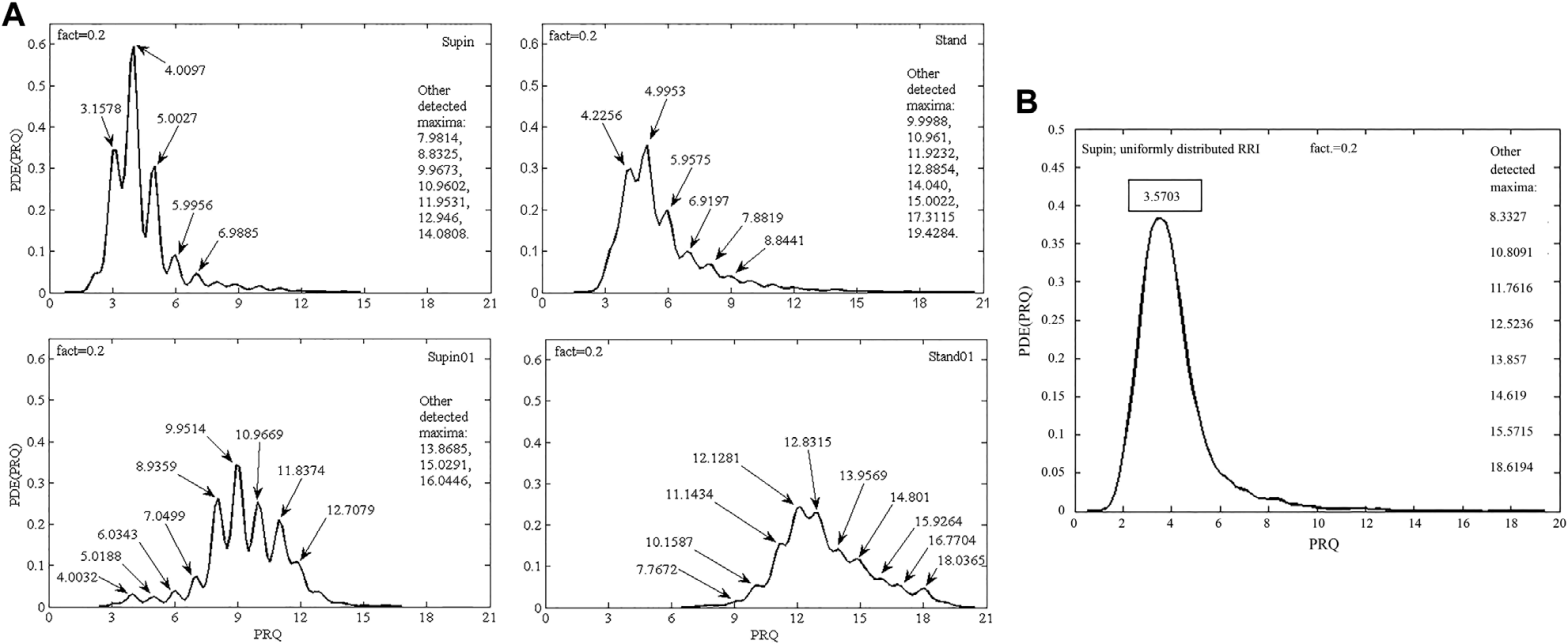
**(A)** PDE distribution profiles of the PRQ parameter for 20 subjects in four physiological states: Supin—supine position with spontaneous breathing, Stand—standing with spontaneous breathing, Supin01—supine position with slow (0.1 Hz) breathing, Stand01—standing with slow (0.1 Hz) breathing; fact—fineness factor. Quantization around integer PRQ values is visible in most local maxima; **(B)** PDE distribution of PRQ parameter for 20 subjects that was calculated from artificial RR intervals (RRI) with uniform distribution (random values within the physiological limits of the RRI parameter) and real BB intervals.

**FIGURE 5:**
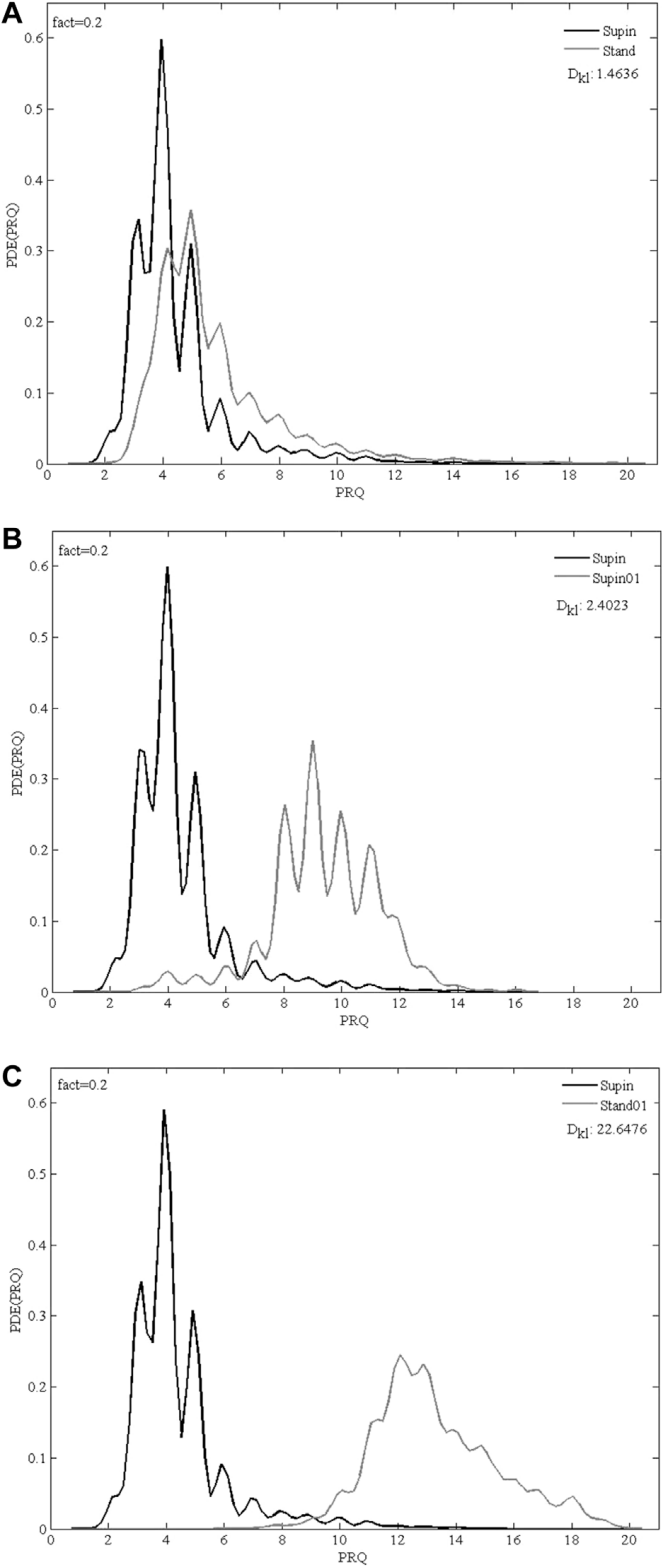
Overlapping PDE distribution profiles of PRQ parameter for 20 subjects in 3 comparison of physiological states: **(A)** Supin-Stand, **(B)** Supin-Supin01, **(C)** Supin-Stand01; Supin—supine position with spontaneous breathing, Stand—standing with spontaneous breathing, Supin01—supine position with slow (0.1 Hz) breathing, Stand01—standing with slow (0.1 Hz) breathing; fact—fineness factor; DKL—Kullback-Leibler divergence (relative entropy).

To check, if the quantization (observed in Supin, Stand, Supin01 in Figure 4A) occurs as a consequence of physiological mechanisms (cardiorespiratory coupling) rather than it is an artifact or bias, we used artificial RR intervals with a uniform probability distribution within the same limits as physiological RRI to calculate the supine PRQ parameter. If our assumption is valid, the quantization of PRQ parameter values should not occur in the PDE distribution under these conditions. The result of this procedure is shown in Figure 4B and supports our choice of methods. Similar results were obtained with reshuffled physiological RR intervals (not shown). Figure 5 displays the size of divergence of PDE distributions in Stand, Supin01, Stand01 in respect to the one in Supin (referent state).

## Discussion

The scope of this work is based on the representation of methodological and interpretative advancements (with respect to Matić et al., 2020) in analysis of cardiorespiratory adaptability in different physiological states. Its outputs are novel insights which have translational component from basic science to applied telemedicine. In general, PRQ quantification has great research potential. The insights regarding our results related to PRQ can be summarized as follows:

• Physiological and clinical significance of PRQ variability indices: results obtained by our new quantification method demonstrated that PRQ variables have greater sensitivity to influence of orthostasis and slow breathing than mean and standard deviation of heart rate and breathing. This indicates that such PRQ quantification could be used for the detection of physiological state changes;
• The main advantage of our approach compared to earlier ways of obtaining PRQ is the continuous and precise calculation of its values, which enabled us to perform a more accurate analysis of PRQ variability;
• New calculation method potentiated insights into fine temporal PRQ variability patterns that are state dependent and that thereby reflect “the state of cardiorespiratory adaptability” (Scholkmann and Wolf, 2019);
• When displayed in minute resolution scale, PRQ mean vs. standard deviation form state specific clusters of values; we hypothesize that these clusters could represent state specific ventilation-perfusion regimes;
• The main highlight of this research is related to PRQ quantization (appearance of integer values in ratio quotient between heart rate and breathing rate); our method of calculation and analysis of probability density estimation (PDE) distributions gave more evident and precise determination of PRQ quants than classical distribution views used in previous research. In fact, by calculating only integer PRQ values, construction of PDE profiles would not be possible. Beside characterization of state dependence of mean and st dev of PRQ (reported for the first time in the states of combination of slow breathing with supination and slow breathing with standing), the PDE distribution of PRQ for the first time registered integer values and the presence of PRQ quantization mechanism in physiological states metabolically and homeodynamicaly different from supine position. Our results show for the first time (to the best of our knowledge) that PRQ quantization exists in variable proportion to “non-integer” PRQ mechanisms even under the influence of slow breathing and orthostasis. Therefore, novel method revealed that PRQ quantization mechanisms make a ubiquitous, constitutive part of cardiorespiratory coupling in supination and in (potentially other) physiological states. It would be of utmost importance to see if PRQ quantization mechanisms remain present (lost or emphasized?) in different cardiorespiratory pathologies and what would be their profile difference with respect to healthy state.

Here we introduce a new method for the precise calculation of both integer and non-integer parts of the RR intervals within a respiratory cycle to accurately and continuously determine instantaneous pulse respiration quotient (PRQ). With our approach, by summing all integer and non-integer parts of RR intervals within a recorded series, the total number of RR intervals is conserved over the whole signal. This is possible because our method preserves each integer and each non-integer part b1and b2 within each respiratory cycle, defined from one inspiration to the next. Although in mathematical terms these PRQ values are rational numbers, we found an accumulation of integer PRQ values, the local maximum appearing over integer PRQ values could be interpreted as PRQ aggregating around that integer.

The results indicate that cardio-respiratory coupling has a certain dynamic that is dependent on specific physiological conditions such as body position and breathing regime and is probably regulated by the activity of the ANS. It is clear from Table 1 and Figure 2 that the supine state is characterized by low values of PRQ. Supine state also is related to increased vagal activity. Our result of mean value of PRQ (=4.8, Table 1) is above the other references specific for supine resting position with spontaneous breathing: 4.1 (Edelhäuser et al., 2013; van Bonin et al., 2014) and 4.2 (Zohdi; Scholkmann and Wolf 2020). It turns out that in our sample interindividual variability elevates the mean value of PRQ, because there are subjects with an average PRQ significantly higher than in the majority of the sample. However, our result of PRQ mean value is below 5.03 and 5.29 that were obtained by Grote et al. (2021). With the duration of the supine state, this decrease becomes more expressed; mean and std dev are significantly lowered (Table 1) which is visually notable in Figure 2 as *narrowing and drop of the PRQ values*. On the other hand, orthostasis (which is usually more sympathetically modulated) and slow breathing induce elevation of PRQ. With respect to Supin, PRQ in prolonged Stand01 increases noticeably. Regarding the results presented in Table 2, it is important to note that the new quantity PRQ appeared to be more sensitive to condition changes (Supin-Stand, Supin-Supin01 and Supin-Stand01) than all the other tested quantities (mRRI, sdRRI, mBBI and sdBBI) (Matić et al., 2020). This qualifies this new measure also for the detection of physiological state changes. The accurate calculation of PRQ, which has been used in medicine earlier (Hildebrandt et al., 1998, 2013) but not continuously calculated, could be of interest for the preparation and performance of space flights with human crew, of sportsmen and non-professional athletes, and of patients with various pathologies *etc.* This is in line with the research of Armañac-;Julián et al. (2021) which pointed out that compared to HRV indices, values of cardiorespiratory coupling (CRC) were more significantly altered in patients with failed weaning, which enabled the authors to differentiate such patients as risky from those that had successful weaning (Armañac-Julián et al., 2021). Superiority of PRQ vs. HRV for the detection of the severity of cardiac pathologies was already described by Weckenmann in 1975 (Weckenmann 1975).

In Figure 3 we report specific forms of clustering of PRQ mean values vs. PRQ standard deviation, the position and shape of which are dependent on the physiological state. Following the sequence Supin-Stand-Supin01-Stand01 we report an opposite tendency of PRQ mean value (increase) and PRQ standard deviation (decrease). Regarding oxygen transport efficiency of cardiorespiratory system, these clusters could represent state specific ventilation-perfusion regimes, closely correlated to increasing metabolic demands of the Supin-Stand-Supin01-Stand01 sequence (Bojić et al., 2020). Regarding the adaptability of the cardiorespiratory system, in our previous study (Matić et al., 2020) we identified the physiological state Stand01 as the state with the most specific CRC in nonlinear domain. PRQ finding supports our hypothesis that Stand01 is the state with the highest potential for cardiorespiratory adaptability (Papaioannou et al., 2011; Matić et al., 2020). In the light of dynamic characteristic of PRQ during this state we could conclude that cardiorespiratory adaptability is most efficient in the state of a highly regulated BBI vs. RRI relation, expressed as a (high) mean value and standard deviation of PRQ (Bojić T et al., 2021). It might be plausible that such graded and a state specific average PRQ value vs. PRQ standard deviation (Figure 3) are necessary preconditions for the state specific cardiorespiratory network adaptability and learning processes initiation and action. In our previous research Stand01 was behaviorally and metabolically the most complex state. It showed the most specific pattern of CR nonlinear coupling, critically important for the adaptability of the cardiorespiratory system (Matić et al., 2020). So, specific results for both nonlinear (Matić et al., 2020) and linear indices in Stan01 indicate that standing and slow breathing in combination generate unique physiological circumstances, a new and unusual situation for the organism’s regulatory system. The peculiarity of this state is that, unlike when lying down, it is unexpected for a subject to breathe slowly while standing. In this sense, it is possible that such a combination of body position and breathing rhythm represents *a provoking (unexpected) stimuli* which induces sympathetic and parasympathetic amplified activity and that as such it could be used as an activation trigger and conditioning excersise of ANS in condtions followed by autonomic disregulation. These results are output from altered (novel) interaction between cardiac (heart rate and blood pressure rate variability), respiratory (0.1 Hz resonant oscillations) and posture (balance and upright meintance) control systems (Siedlecki et al., 2022). Novel, unexpected stimuli are sometimes needed for reactivation of faltered neural feedback systems. Stable, state specific (Figure 3) continuous PRQ regulation could be the prerequisite, a sort of sublime temporal frame of the body’s oxygenation regime for the activation of the specific cardiorespiratory adaptability patterns (Matić et al., 2020). Further studies of PRQ relation with CRC nonlinear parameters with learning paradigms (i.e., characterization of memory patterns after daily exercise of standing with slow 0.1 Hz breathing during a certain time period vs. standing with spontaneous breathing) are necessary for the evaluation of this hypothesis. Quantization with a preference for integer values ​​is noticeable on the histogram representations (in supine position) published in different independent studies (Moser et al., 1995; Kotani et al., 2002; Bartsch et al., 2007; Scholkmann and Wolf, 2019; Scholkmann, Zohdi, and Wolf, 2019). Their PRQ histograms indicate a pronounced accumulation of PRQ integer values HR:RR = m:1 (Moser et al., 1995; Kotani et al., 2002; Bartsch et al., 2007; Scholkmann and Wolf, 2019) and especially during sleep (Grote et al., 2021). We also noticed the same accumulations in the supine condition (Supin) with an affinity towards the integer ratio 4:1. In other states (Stand, Supin01, Stand01) a shift towards higher values was observed. Also, it can be seen that different states result in specific forms of PRQ value distributions. This is in line with the research of Scholkmann et al. in which the emphasis was on the analysis of histogram shapes (F. Scholkmann, Zohdi and Wolf 2019). The novelty of our research is that, by the calculation of PRQ non-integer values and its PDE distribution, we obtained PRQ state specific quantization behavior, not only for rest (Supin), but also under the influence of changes in body position and slow breathing. Appearance of PRQ quantization over a broad range of integers is most probably the result of inter-individual variability within the group. Physiological origin of integer peaks of PDE distributions was confirmed by the PDE analysis using synthetic RRI per real BBI, from which we obtained a “smooth” distribution over all values of PRQ, and where local maxima of integer PRQ values did not show (Figure 4). Thus, the physiological regulation responsible for PRQ quantization is not an on-off phenomenon and not an exclusive property of the supine condition (Moser et al., 1995; Scholkmann and Wolf, 2019). Among all four studied states, in Supin state, corresponding to a high vagal activity, the relation of PRQ quantization vs. the PRQ non-integer generating mechanisms is most emphasized. In other states PRQ mean value is shifted towards higher values (Stand, Supin01, Stand01), but quantization is less pronounced. Visual inspection shows that PRQ quantization is more determined by body posture (Supin vs. Stand) than by the change of the breathing regime (Supin vs. Supin01). PRQ quantization is least visible in Stand01, where slow paced breathing regime and standing position manifest synergetic suppression. PRQ quantization seems to be an ubiquitous phenomenon for all four tested physiological states, with a state dependent relative contribution of regulation to the respective amount of quantization. One of the mechanisms proposed in the literature as opponent to PRQ quantization mechanism(s) is respiratory sinus arrhythmia, maximally expressed during physiological quiescence of parasympathetic *modulation* predominance with sympathetic *modulation* withdrawal on HR (Kotani et al., 2002; Bartsch et al., 2007). Our results are not in line with this observation. However, by our approach, the influence of respiratory sinus arrhythmia on PRQ is effectively removed, because all RR variations within each BB interval are averaged out. PDE analysis of PRQ values shows that orthostasis (sympathetic predominance with parasympathetic withdrawal on HR regulation) has a suppressive effect on PRQ quantization. Joined sympathetic and parasympathetic activity (Stand01) has, apparently, additive suppressant effect on PRQ quantization mechanism. It is reasonable to hypothesize that sympatho-vagal relation, specific for the resting state and sleep (vagal dominance, sympathetic withdrawal, Levy, et al., 1996) allows the emergence of emphasized PRQ quantization. With regards to this, our results reflect the ubiquitous presence of adaptive effect of ANS towards the integer harmonization of heart rhythm and respiration. This effect can be especially noticed on the summary diagram of PRQ dynamics in Figure 2 and in Table 3, which contains the minute mean values ​​of PRQ. Namely, after approximately 14 min at supine position with spontaneous breathing (Supin), a very pronounced accumulation of PRQ values ​​around number four is notable—*the drop and narrowing of PRQ flow*, as we mentioned. Apparently, there is an enhancement of quantization by staying in a supine position for a longer time. Scholkman and Wolf (2019) and Grote et al. (2021) mention something similar during sleep - a decrease in PRQ variability and fixation to the whole-number ratio 4:1 (Scholkmann and Wolf 2019). Main factor of this result in sleep is transition from standing to supine position and prolonged staying in supine position in which decrease of heart rate takes place, whereas decrease of respiration rate originates from transition from wakefulness to sleep (von Bonin et al., 2014). This behavior can be characterized as a homeostatic amplification (mean value steady regulation) that overcomes the homeodynamic principle; or in other words, this is the energetically optimal coupling pattern between heart rhythm and respiration, an effect of activating the physiological mechanisms of the organism’s recovery during the rest (prolonged stay in a supine position). It is plausible that PRQ quantization, as an intrinsic property of the cardiorespiratory regulatory network (Kotani et al., 2002; Bartsch et al., 2007) represents a safe, low energy cost state for CPC and maintains the ventilation/perfusion ratio especially while the higher order autonomic networks perform reparatory and regenerative processes as in sleep (Bojic, 2003; Grote et al., 2021), rest (Moser et al., 1995) or recovery after surgery in which increased vagal activity has great significance, due to its immune-modulating properties (Grote et al., 2019). Thus, prolonged stay in supine position (up to 30 min) during day might be beneficial for well-being, e.g., alertness and learning ability (van Bonin et al., 2014).

A breathing regime of 0.1 Hz drives PRQ relation towards the value of 10:1 (Table 1. Supin01–9.41 ± 1.21, Stand01–13.48 ± 1.66; Figure 2. Main (highest) quantization peak in Supin01–9.9514). It should be clear that 10:1 value of PRQ mostly result from experimental setting in which the breathing cycle is 10 s, while average RRI take values close to 1 s (Table 1). *Therefore, the 10:1 PRQ relation of slow breathing is not completely analogous to 4:1 PRQ relation of spontaneous breathing, since the latter is present without conscious adjustments of the breathing rate*. What is evident as a physiological result is that 0.1 Hz slow breathing induce an increase of RRI variability (for 45.7% in respect to spontaneous breathing regime, sdRRI, Table 1). It could also be the manifestation of a resonance effect (synchronization) achieved by respiratory induced increase of vagal modulation (Gerritsen and Band 2018; Sevoz-Couche & Laborde 2022). This regime results with phase synchronization between heart rate and breathing (just as with baroreceptor resonant frequency (increased baroreflex sensitivity, Zautra et el 2010; Steffen et al., 2017; Pagaduan et al., 2019; Sevoz-Couche and Laborde 2022) which is paired with a maximal gas exchange (Lehrer et al., 2020) and higher values of arterial blood oxygenation (Bilo et al., 2012) but with higher metabolic cost due to extensive activation of the “respiratory pump”. Hypothetically, if 0.1 Hz training of breathing regime stimulates neural network learning and positioning of PRQ around 10:1, we presume, in accordance with the literature (Bernardi et al., 1998; 351; Russo et al., 2017) that this state would be characterized by higher arterial blood oxygen saturation, higher oxygen consumption, and therefore with positive consequences on overall oxidative metabolic processes.

The study of Bernardi et al. (1998) of the effect of breathing rate on oxygen saturation and exercise performance has confirmed this by measuring arterial oxygen saturation during spontaneous breathing and paced at 15, 6 and 3 breaths per min, in rest and exercise, in healthy subjects and in chronic heart failure patients. Slow breathing at 6 breaths per min (0.1 Hz) was found to be optimal for improving alveolar ventilation and reducing functional dead space in both investigated groups, in terms of increased arterial oxygen saturation and low respiratory effort. Follow-up of chronic heart failure patients who continued the practice of slow breathing, showed increased exercise performance and motivation (Bernardi et al., 1998). Long term beneficial influence of slow breathing on oxygen saturation, perfusion index and PRQ and their inter-relatedness was indicated in study of Xia (2022).

Therefore, analogous to the value of four that appears in Supin, one characteristic (resonant) value of PRQ could have reinforcing influence on synchronization in supine position with slow breathing as well (Supin01). So far, we detected several quantization values in Stand and Supin01 states, but we do not know is their effect similar to value four of Supin state. It seems that slow breathing enhances the tendency towards more integer values, which can be attributed to different resonance effects of slow breathing (Sevoz-Couche and Laborde 2022), as well as the simple fact that BB intervals are artificially extended to 10 s.

Recent research showed that vagal activity is more pronounced during autoregulative periods than sympathetic drive. In Stand, a vagal withdrawal occurs that enables sympathetic drive to be more dominant (Maggioni et al., 2020). Therefore, general cardiorespiratory autonomic responsiveness (adaptability) (Malik et al., 2019) is mostly based on vagal reactivity (Grant et al., 2012; Maggioni et al., 2020). Our observations agree with the statement that “parasympathetic withdrawal reactive to orthostatic challenge”, represent “an essential marker of healthy autonomic reactivity” (Maggioni et al., 2020).

In contrast to the supine-induced relative homeostasis, in Stand, Supin01, Stand01 a homeodynamic regulation (an increase of variability, Table 1) is more pronounced.

In the Stand01 state a marked increase in the mean value and standard deviation of PRQ occurs, but without a prominent integer quantization, so we could talk about the combined inhibitory effect of slowed breathing (vagal amplification) and standing (sympathetic amplification) on PRQ quantization. Stand01 was the only state in which variability of PRQ increased significantly (Table 2: sdPRQ rising by 34.4% in respect to Supin). The influence of slow breathing on the rise of RRI variability (sdRRI) in Stand01 was significant (for 13.04% in respect to Supin), but not as pronounced as in Supin01 (by 45.7% in respect to Supin).

In our PDE profiles (Figure 4) the quantization may assume all values ​​of natural numbers up to some maximum value. Using a surrogate signal with uniformly distributed or reshuffled (not shown) RR intervals we could show that this is not a technical artifact: All local maxima either disappear, or acquire non-integer PRQ values, except the one close to PRQ ≈4:1, under these conditions. As PDE (PRQ) and PDE (BBI) are similar in shape, PDE (RRI) appears as a sharp peak positioned over RRI ≈1:1 (not shown). PRQ quantization therefore is additional mark of CRC synchronization, because if heart beat and respiration were completely desynchronized, i.e., uncorrelated, then the inspirium nadir of a BB interval would partition the corresponding RR interval at a random location, making non-integer PRQ values ​​as probable as integers, and quantization peaks would not occur, which is not the case according to our study. The reason for this could be that the systolic and diastolic phases are the times of feed-back for the higher regulatory networks (i.e., instantaneous systemic arterial pressure and oxygenation monitoring in systole, or cardiac chamber filling, venous pressure regulation, cardiac humoral regulation of body fluids in diastole, *etc.*). The integration of such complex information, for example, obtained during systole, mediated by baro- and chemo-receptors up to higher cardiorespiratory regulatory centers, would be disturbed by concomitant chemo-neuro-mechanical effects of the inspirium. This hypothesis is further supported by the fact that CRC is bidirectional and that cardiac impact on respiration is prevalently mediated by baroreceptors (i.e., on beat-to-beat basis; Dove and Katona, 1985; Jung R and Katona, 1990; Kotani et al., 2002).

It is also likely, that the coupling of the inspiratory onset to the cardiac cycle (Moser et al., 1995) improves the oxygenation of blood in the lung: Just 150 msec after the electric systole (R-peak), most inspirations in a healthy subject start. This is approximately the delay that the heart needs to start the mechanical systole (electromechanical coupling) which than moves the blood. Blood from the right ventricle starts to perfuse the lung tissue and at the same time inspiration starts and the lung is filled with air. Thoracal pressure is reduced due to the inspiration which helps to perfuse the lung with air and blood. Due to the cardio-inspiratory coupling the likeliness of whole number ratios between heart beat and respiration is increased, which makes a whole number PRQ an interesting indicator of good efficiency of blood oxygenation.

Regarding the reference value of minimal relative entropy (D_KL_ = 0) which indicates that the two distributions have the same quantity of information (Kullback and Leibler, 1951), the results obtained in our research (Figure 5) indicate that standing and slow breathing produces a significant divergence in PDE distributions (D_KLSupin-Stand_ = 1.4636, D_KLSupin-Supin01_ = 2.4023), while their joint influence exerts much greater change of PDE distribution (D_KLSupin-Stand01_ = 22.6476). This joint influence of slow breathing and orthostasis is not a simply linear additive effect, since D_KL_ as a measure does not fulfill requirements for a metric distance, as it does not adhere to the triangle inequality rule. Thus, it is likely that the combined orthostatic and respiratory stimuli of Stand01 produce a certain nonlinear amplifying effect which should be further explored in context of different demanding cardiorespiratory regimes.

## Data Availability

The raw data supporting the conclusions of this article will be made available by the authors, without undue reservation.

## Footnotes

1
https://www.ibm.com/support/pages/post-hoc-comparisons-kruskal-wallis-test.
